# On Certain Gas-Forming Bacteria of the Alimentary Canal, Their Fate in the Stomach, and Their Reaction on Different Foods

**Published:** 1886-04

**Authors:** W. D. Miller


### Independent Practitioner.

VOL. VII. APRIL, 1886. No. 4.

### Original Communications.

### ON CERTAIN GAS-FORMING BACTERIA OF THE ALIMENTARY CANAL, THEIR FATE IN THE STOMACH, AND THEIR REACTION ON DIFFERENT FOODS.

BY DR. W. D. MILLER.

(Continued from the March number.)

In a previous number of this journal I made especial reference to five kinds of bacteria, which are charcterized by the very considerable quantities of gas which they produce in starchy and saccharine substances, and further, by the marked resistance which they possess to the action of the gastric juice.

In view of the great importance which a thorough knowledge of the physiology of these micro-organisms has in the therapeutics of many disorders of the stomach, and especially in the dietetic treatment of such troubles, the results of a series experiments made in connection with this subject may not be without value.

Two questions in particular appear to me to require a solution: 1st. Are the micro-organisms which are taken into the stomach at any meal passed out or devitalized before the recurrence of meal-time, or does the stomach, of dyspetics in particular, contain fungi at all times? 2d. In what manner is the quantity of gas generated by these fungi influenced by the kind of food taken?

Respecting the first question, practical experience would incline us to the belief that the diseased or impaired stomach contains micro-organisms at all times, otherwise how are we to explain the fact that many patients almost immediately after taking starchy or saccharine foods are troubled with the distention of the stomach from formation of gas? I have demonstrated by various experiments that those fungi alone which may be swallowed with a piece of bread are certainly not capable of bringing about this distention. Nevertheless, it appeared desirable to test the correctness of this supposition experimentally; and since the proper experiments could be performed on the human subject only with great difficulty, I chose dogs as the subjects of experiments, as they have a digestive process very similar to that of human beings. Up to the present but six dogs have been experimented upon, but as the results were the same in every case, they are entitled to consideration.

Four of these dogs, all healthy, were fed for two days on a mixed diet of meat, bread, milk and sugar, richly infected with the gas-producing bacteria referred to. In every case diarrhœa made its appearance in from 30 to 40 hours, and in two cases distention of the stomach was very marked. After the last feeding they were muzzled to prevent the possibility of their taking anything into the stomach, and killed after 2½, 6, 8 and 9 hours respectively. In every case, except the last, the four kinds of fungi administered were found in the stomach in great numbers, also in the large intestines; but few were present in the duodenum. In two cases the reaction of the contents of the intestines was strongly sour throughout, in one case slightly sour, and in one only was the reaction neutral or slightly alkaline. The remaining two dogs were treated in the same manner, except that the bread and sugar were omitted; the result was the same as above, only more marked. One of the dogs had profuse diarrhœa in 15 hours, and the intestines, six hours after feeding, were distended with gas, and the reaction strongly sour. In both cases the fungi were found in large numbers in the stomach, as well as in the intestines.

It appears from these experiments that these fungi can maintain their existence, even in the stomach of dogs, for 8 or 10 hours, and can thereby give rise to disturbance in the intestinal canal, and when we take into account the fact that the gastric juice of the dog is 1½ times as strong as that of the healthy human subject, and from 1½ to many times as strong as that of the dyspeptic or stomach-ailing, there is little room left for doubt that living fungi are constantly present in the stomach, and that many chronic troubles of the stomach are due, not alone to the micro-organisms which are at each meal taken in along with the food, but more particularly to those which are already in the stomach at the beginning of a meal.

It is, consequently, of greater importance to sterilize the stomach before eating than to sterilize the food itself. For example: What is the use of repeatedly boiling milk to free it from every single germ, and then taking it into a stomach containing vastly more fungi than were in the milk in the beginning? It will furthermore be readily seen that the time for administering antiseptics is not after a meal, or upon the full stomach, but upon an empty stomach, about 10 or 15 minutes before meal-time. In order to determine what action these fungi may have upon the digestive process when the conditions present are such as to permit of their development, I swallowed a full grown test-tube culture of the *micrococcus aerogenes* immediately after a meal, consisting mainly of bread, potatoes and milk. A very disagreeable feeling of distention in the stomach and bowels appeared after about 1¼ hours, and annoyed me the whole of the next day; after 30 hours I was attacked with diarrhœa, which I aborted by taking 60 drops of *concentrated hydrochloric acid* after a light breakfast. The effects of the fungi did not entirely disappear for some days, and even on the fifth day I still found them in my fæces.

I attempted to effect a solution of the second question by the following experiments: I mixed 2.0 grams (30 grains) of the different kinds of food with 3.0 of saliva, added 5.0 grams beef-extract peptone-gelatine, which had been infected with *bacteria aerogene* (as I simply chewed up the food to be tested and mixed it with an equal quantity of the same gelatine). Each mixture was then poured into a separate test-tube, the level marked and placed at a temperature of 22° C. The amount of gas generated in the different tubes would be measured by the rise in the surface of the mixture. After a series of 19 experiments I succeeded in getting together a table of comparative values, in which bread is used as the unit of comparison.

As expected, it was found that the carbo-hydrates are particularly characterized by the production of large quantities of gas, whereas from the albuminous substances only a trace, or no gas at all, is produced. Among the ordinary articles of diet, bread and potato occupy a prominent place as gas-producers, and should be as much as possible avoided by the dyspeptic.

Of those foods which do not appear in the table, and which produce large quantities of gas, I may mention all kinds of sweet desserts, cakes, omelettes, pears, sweet apples, grapes, etc., etc. Cranberries and prunes produce no gas, because they do not furnish a favorable medium for the development of bacteria; if they are mixed with meat, however, a very rapid development takes place, accompanied by a production of gas.

According to these experiments, a diet for stomach-ailing patients, which should be followed by no production of gas, distention of the stomach, sour eructation, etc.,, etc., may consist of meat, eggs, fish, spinach, cheese, fresh lettuce, and small quantities of endives and sour cranberries.

The administration of an antiseptic before, and a digestive during or immediately after the meal, will materially aid the process of digestion.

I have had abundant opportunity to observe the happy action of the above diet on one who has for years been troubled with indigestion, arising from abnormal fermentation in the stomach.

I have thought that the above experiments might deserve a place in a dental journal, because certainly no professional man is more relied upon to maintain the integrity of the alimentary canal than the dentist, a healthy mouth being the first requisite thereto. I have, however, omitted the Morphology, Physiology, etc., of the micro-organisms under consideration, which have received the names *Bacterium aerogenes, I, and II.*, *Bacillus aerogenes*, *Micrococcus aerogenes*, and *Helicobacterium aerogenes.*

